# Synthesis and Evaluation of Thiochroman-4-One Derivatives as Potential Leishmanicidal Agents

**DOI:** 10.3390/molecules22122041

**Published:** 2017-11-29

**Authors:** Esteban Vargas, Fernando Echeverri, Iván D. Vélez, Sara M. Robledo, Wiston Quiñones

**Affiliations:** 1 Química Orgánica de Productos Naturales, Instituto de Química, Facultad de Ciencias Exactas y Naturales, Universidad de Antioquia, calle 70 No. 52-21, Medellin A.A 1226, Colombia; esteban.vargasc@udea.edu.co (E.V.); fernando.echeverri@udea.edu.co (F.E.); 2 PECET-Facultad de Medicina, Universidad de Antioquia, calle 70 No. 52-21, Medellin A.A 1226, Colombia; ivan.velez@udea.edu.co

**Keywords:** *Leishmania*, thiochroman-4-ones, thiochromones, thioflavones, vinyl sulfone

## Abstract

The S-containing heterocyclic compounds benzothiopyrans or thiochromones stand out as having promising biological activities due to their structural relationship with chromones (benzopyrans), which are widely known as privileged scaffolds in medicinal chemistry. In this work, we report the synthesis of 35 thiochromone derivatives and the in vitro antileishmanial and cytotoxic activities. Compounds were tested against intracellular amastigotes of *Leishmania panamensis* and cytotoxic activity against human monocytes (U-937 ATCC CRL-1593.2). Compounds bearing a vinyl sulfone moiety, **4h**, **4i**, **4j**, **4k**, **4l** and **4m**, displayed the highest antileishmanial activity, with EC_50_ values lower than 10 μM and an index of selectivity over 100 for compounds **4j** and **4l**. When the double bond or the sulfone moiety was removed, the activity decreased. Our results show that thiochromones bearing a vinyl sulfone moiety are endowed with high antileishmanial activity and low cytotoxicity.

## 1. Introduction

Cutaneous leishmaniasis (CL) is an anthropozoonotic disease caused by parasites of the genus *Leishmania*, and is transmitted through the bite of female insects of the genus *Phlebotomus* and *Lutzomyia* [1]. According to the World Health Organization, the disease is widely distributed around the world, with 310 million people at risk and about one million new cases occurring annually. The highest numbers of cases of CL are concentrated in 10 countries (Afghanistan, Algeria, Brazil, Colombia, Iran, Pakistan, Peru, Saudi Arabia, Syria and Tunisia) [2]. Although most cases do not become deadly, the disease produces severe skin lesions that significantly decrease the quality of life of those who suffer from it. Due to a lack of vaccines, disease control measures are based on chemotherapy. The current therapeutic treatments are associated with several drawbacks affecting the adequate management of the disease, such as severe side effects [1], likelihood of not ending treatment mainly in rural areas due to the complexity of treatment [3,4,5], high cost and emerging resistance [3,4]. Therefore, there is an urgent need to find new alternatives to treat CL through different mechanisms of action to facilitate the handling of cases, given the poor therapeutic response to current available medications.

Chromones (1,4-benzopyrone) are known as privileged scaffolds [6,7,8] in medicinal chemistry due to their wide range of biological activities, displayed, among others, as antiprotozoal [9,10], antioxidants [11] or anticancer agents [12,13,14]. The S-containing heterocyclic compounds, benzothiopyrans or thiochromones, have been significantly less exploited, most certainly because they are not naturally occurring compounds; from a bioisoterically point of view, thiochromones could serve as a rational modification for the optimization of chromone bioactivity.

In this work, the benzothiopyran scaffold (thiochroman) was analyzed as a potential source of leishmanicidal agents. These compounds were prepared starting from thiophenol or its derivatives, which act as nucleophiles to α,β-unsaturated carboxylic acids. Then, cyclization to give benzothiopyrans was achieved using a process catalyzed with sulfuric acid, methanesulfonic acid or oxalyl chloride, followed by tin chloride. After construction of benzothiopyran scaffold, oxidation of sulfur atoms to sulfones and dehydrogenation to produce an α,β-unsaturation to the carbonyl group were made. Thus, 34 compounds with structural changes in different parts of the benzothiopyran moiety were synthesized. The leishmanicidal activity and cytotoxicity were tested for all compounds, and structural activity relationships were proposed.

## 2. Results

### 2.1. Synthesis

**Scheme 1:**
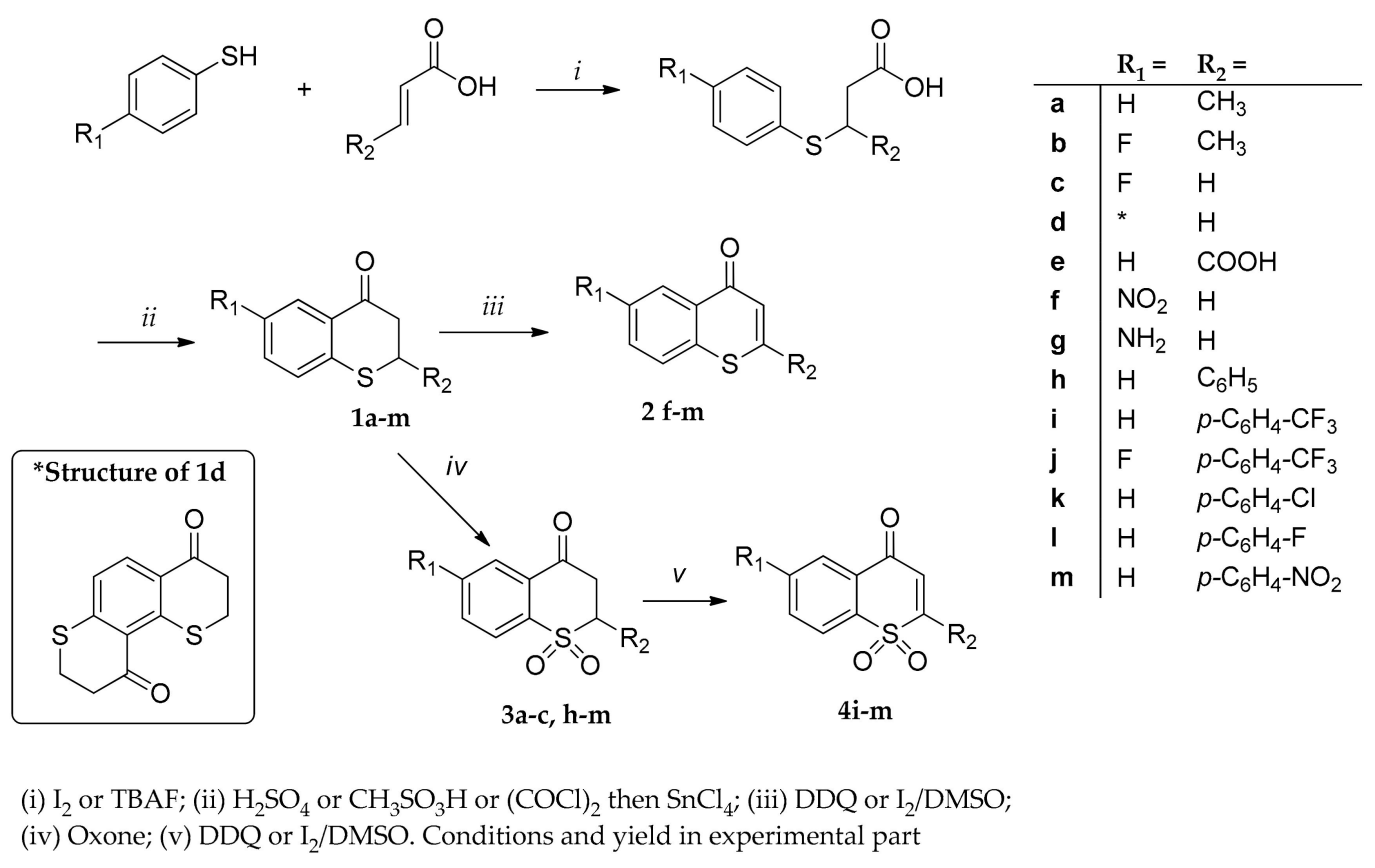
Synthesis of thiochroman-4-one derivatives.

Thiochroman-4-ones have been generally synthesized using a substitution reaction of thiophenol with β-halopropionic acids or β-butirolactones to give 3-(phenylthio)-propanoic acids; then, upon intramolecular Friedel-Crafts acylation, thiochroman-4-ones were produced in moderate yields [15,16,17,18]. Another reaction involves only one step, using direct reaction between α,β-unsaturated acids (acrylic acid derivatives) and thiophenol in the presence of methanesulfonic acid, but the yields were low [19]. In this work, the synthesis of 3-(phenylthio)-propanoic acids was achieved through a catalyzed reaction, either with iodine or tetrabutylammonium fluoride (TBAF) in a solventless way (Scheme 1), in high yields [20]; after that, compounds were cyclized by reaction with sulfuric or methanesulfonic acid in moderate yields.

The cyclization of 3-aryl-3-(phenylthio)-propanoic acid failed to give 2-phenylthiochroman-4-one (thioflavanone) under different reaction conditions. Thus, strong dehydrating agents, such as sulfuric, methanesulfonic or p-toluenesulfonic, only gave trace amounts of the expected product; additionally, formation of the respective acid chloride, followed by intramolecular Friedel-Crafts acylation with aluminum trichloride, also failed. Finally, the synthetic procedure was performed according to Bates and Li [21], and thioflavanone was prepared from the acid chloride of 3-phenyl- 3-(phenylthio)-propanoic acid using tin(IV) chloride as a Lewis acid catalyst, with moderate yields. The same method worked well for the preparation of other thioflavanones.

Oxidation of the thioether (sulfide) group of the thiochromanones or thioflavanones to sulfones were carried out using an excess of Oxone^®^ in a mixture of water/ethanol (3:1). The dehydrogenation reactions were explored using different methods, following similar reports of treatment with DDQ [22], PCl_5_ [23] or a mixture of Iodine/DMSO [22] with moderates yields.

### 2.2. Antileishmanial and Cytotoxic Activities

Thirty-four synthesized compounds, i.e., thiochroman-4-ones and analogues, were evaluated for their in vitro antileishmanial and cytotoxic activities, following the methods of Pulido et al. [24]. Amphotericin B was used as a control (Table 1) with EC_50_ and LC_50_ values of 0.32 μM and 39.6 μM respectively.

## 3. Discussion

**Figure 1:**
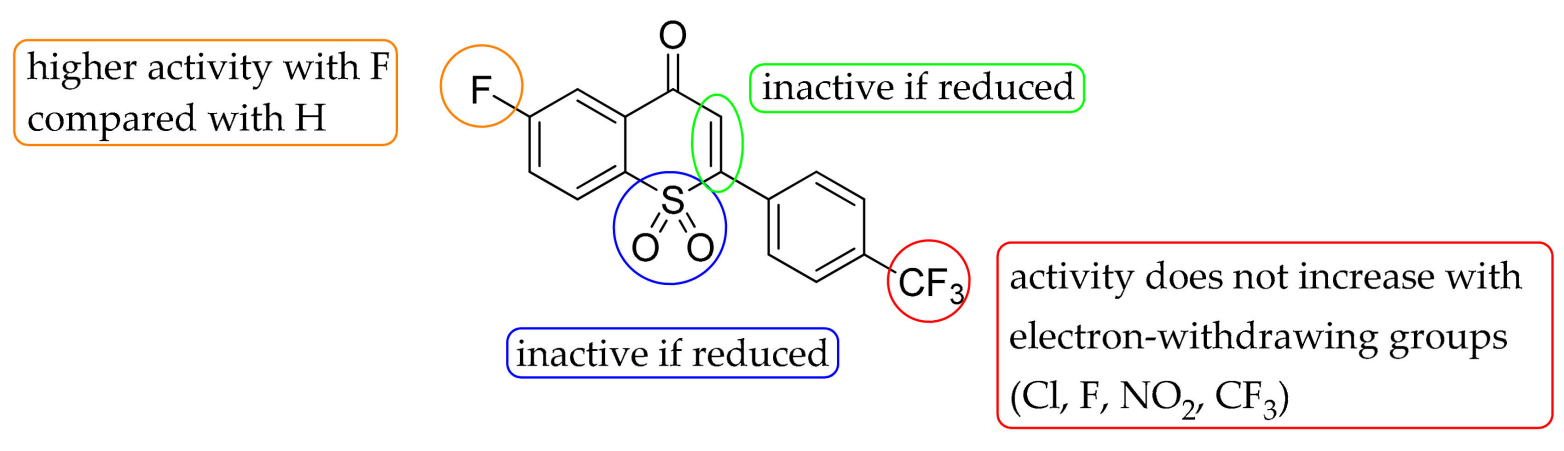
Structure activity relationships of thiochroman-4-one derivatives.

Initially, modifications were made in thiochroman-4-one compounds (**1a**–**m**) to identify structure-activity relationships (Figure 1). The substitution with electron-withdrawing groups in the phenyl ring, dehydrogenation to yield a double bond between C2-C3 (**2f, 2i**–**m**) and sulfones (**3a**–**c, 3h**–**m**) did not improve antileishmanial activity. However, when sulfones were dehydrogenated to produce the corresponding vinyl sulfones (**4h**–**m**), compounds showed high antileishmanial activity, evidenced by EC_50_ values < 10 μM, low cytoxicity to human macrophages U-937 (LC_50_ values > 561 μM) and a high index of selectivity (IS ≥ 87).

Fluorine substitution at C-6 position caused an increase in the leishmanicidal activity in comparison to hydrogenated compound. In fact, compound **4j** was the most active (EC_50_ = 3.23 μM) and selective compound, with an IS value of 174, even higher than the reference drug, amphotericin B. It is interesting to note that all compounds bearing the vinyl sulfone moiety displayed high antileishmanial activity and low cytotoxicity. These types of compounds have been reported in a wide range of biological activities, including neuroprotective [25], antiparasite [26,27] and inhibitor of HIV-1 integrase enzyme [28]. The generally-accepted mechanism of action of vinyl sulfones involves their ability to inhibit cystein proteases by a nucleophilic attack on the β position to the sulfone [29]. However, further studies are required to identify the mechanism of action of thiochromenes bearing vinyl sulfones to optimize antileishmanial activity.

## 4. Materials and Methods

### 4.1. Chemistry

#### 4.1.1. General

All commercially available reagents and solvents were obtained from commercial suppliers and used without further purification. The reaction progress was monitored with thin layer chromatography on silica gel TLC aluminum sheets (60F_254_, Merck, Darmstadt, Germany). The melting points were determined using a Mel-Temp apparatus (Electrothermal, Staffordshire, UK) and are uncorrected. FTIR spectra were obtained on a Bruker Alpha FTIR spectrometer (Bruker Optic GmbH, Ettlingen, Germany. ^1^H and ^13^C nuclear magnetic resonance (NMR) spectra were recorded using Bruker DRX 300 spectrometer (Bruker Bio-Spin GmbH, Rheinstetten, Germany) operating at 300 MHz for ^1^H and 75 MHz for ^13^C. Chemical shifts were reported relative to internal tetramethylsilane (δ 0.00 ppm) for ^1^H, and CDCl_3_ (δ 77.0 ppm) for ^13^C. HRMS was obtained using Q-TOF quadrupole/ orthogonal spectrometry (Waters, Milford, MA, USA) in either negative (reported as [M − H]^−^) or positive mode (reported as [M + H]^+^).

#### 4.1.2. Synthesis of Thiochroman-4-ones

*2-Methylthiochroman-4-one* (**1a**). To a mixture of crotonic acid (860 mg, 10 mmol) and thiophenol (1.650 g, 15 mmol) was added I_2_ (20 mol %, 255 mg, 1 mmol) and the mixture was stirred at room temperature for 12 h. After completion of the reaction (monitored by TLC), a cold saturated sodium thiosulfate solution (20 mL) was added and extracted with dichloromethane (2 × 50 mL); then, combined organic layers were mixed with a saturated solution of sodium bicarbonate and extracted to remove the unreacted starting material. The water layer was acidified with hydrochloric acid (10% aq) and extracted with dichloromethane (3 × 40 mL). The combined organic layers were dried over Na_2_SO_4_, evaporation of the solvent under reduced pressure afforded 1.962 g (86%) of the desired addition product. After, 200 mg (1.0 mmol) of this product were cooled down to 0 °C in an ice bath and 3.0 mL of concentrated sulfuric acid was added; the reaction mixture was stirred for 30 min, and, after that, the ice bath was removed allowing the reaction mixture to warm to room temperature for another 2 h under continuous stirring. The reaction was quenched with ice and the mixture was extracted with dichloromethane (3 × 25 mL). The combined organic layers were washed once with water, followed by addition of a saturated NaHCO_3_ solution. The combined organic layers were dried over Na_2_SO_4_, and concentrated under reduced pressure. The residue was purified by column chromatography over silica gel using hexane:EtOAc (9:1) as eluent, to give 137 mg (75%) of pure **1a** as a yellowish oil. ^1^H-NMR (300 MHz, CDCl_3_) δ 8.08 (d, *J* = 8.0 Hz, 1H), 7.38 (t, *J* = 7.5 Hz, 1H), 7.25 (d, *J* = 7.8 Hz, 1H), 7.16 (t, *J* = 7.6 Hz, 1H), 3.74–3.53 (m, 1H), 2.98 (dd, *J* = 17.6, 8.8 Hz, 1H), 2.84–2.66 (m, 1H), 1.43 (d, *J* = 6.8 Hz, 3H). ^13^C-NMR (75 MHz, CDCl_3_) δ 194.9, 141.9, 133.7, 130.1, 129.1, 127.65, 125.1, 48.0, 36.5, 20.6. IR ν: 2964, 1679, 1587. HRMS (ESI) calculated for C_10_H_11_OS [M + H]^+^ 179.0525, found 179.0536.

*6-Fluoro-2-methylthiochroman-4-one* (**1b**). To a mixture of crotonic acid (172 mg, 2 mmol) and 4-fluorothiophenol (385 mg, 3.0 mmol) was added I_2_ (20% mol, 52 mg, 0.2 mmol) and the mixture was stirred at room temperature for 12 h. After completion of reaction (monitored by TLC), a cold saturated sodium thiosulfate solution (20 mL) was added and extracted with dichloromethane (2 × 25 mL); the combined organic layers were mixed with a saturated solution of sodium bicarbonate and extracted to remove the unreacted starting material. The water layer was acidified with hydrochloric acid (10% aq) and extracted with dichloromethane (3 × 25 mL). The combined organic layers were dried over Na_2_SO_4_, evaporation of the solvent under reduced pressure afforded 200 mg (94%) of the addition product. Thus, compounds were cooled down to 0 °C in an ice bath and 2.0 mL of concentrated sulfuric acid was added and the reaction mixture was allowed to warm to room temperature for 2 h with continuous stirring. The reaction was quenched with ice and the mixture was extracted with dichloromethane (3 × 25 mL). The combined organic layers were washed once with water, followed by saturated NaHCO_3_ solution. The combined organic layers were washed with brine, dried over Na_2_SO_4_, and concentrated under reduced pressure. The residue was purified by column chromatography over silica gel using hexane:EtOAc (9:1) as eluent to give 128 mg (64%) of pure **1b** as a yellowish oil. ^1^H-NMR (300 MHz, CDCl_3_) δ 7.84–7.72 (m, 1H), 7.39–6.97 (m, 2H), 3.78–3.47 (m, 1H), 3.07–2.95 (m, 1H), 2.80–2.68 (m, 1H), 1.43 (d, *J* = 6.9 Hz, 3H). ^13^C-NMR (75 MHz, CDCl_3_) δ 194.2, 160.7 (d, *J_C–F_* = 246 Hz), 137.6, 132.3, 129.7 (d, *J_C–F_* = 7.0 Hz), 121.8 (d, *J_C–F_* = 23.1 Hz), 115.4 (d, *J_C–F_* = 22.8 Hz), 47.9, 37.0, 20.7. IR ν: 2967, 1684, 1602. HRMS (ESI) calculated for C_10_H_10_FOS [M + H]^+^ 197.0431, found 197.0443.

*6-Fluorothiochroman-4-one* (**1c**). To a mixture of acrylic acid (700 μL, 720 mg, 10 mmol) and 4-fluorothiophenol (1985 mg, 15 mmol) was added I_2_ (20% mol, 760 mg, 3 mmol) and the mixture was stirred at 50 °C for 24 h. After completion of reaction (monitored by TLC), a cold saturated sodium thiosulfate solution (30 mL) was added and extracted with dichloromethane (2 × 25 mL); the combined organic layers were mixed with a saturated solution of sodium bicarbonate and extracted to remove the unreacted starting material. The water layer was acidified with hydrochloric acid (10% aq) and extracted with dichloromethane (3 × 50 mL). The combined organic layers were dried over Na_2_SO_4_, evaporation of the solvent under reduced pressure afforded 1150 mg (64%) of the desired addition product. The product was cooled down to 0 °C in an ice bath and 3 mL of concentrated sulfuric acid was added and the reaction mixture was allowed to warm to room temperature for 2 h with magnetic stirring. The reaction was quenched with ice and the mixture was extracted with dichloromethane (3 × 50 mL). The combined organic layers were washed once with water, followed by saturated NaHCO_3_ solution. The combined organic layers were washed with brine, dried over Na_2_SO_4_, and concentrated under reduced pressure. The residue was purified by column chromatography over silica gel using hexane:EtOAc (9:1) as eluent to give 570 mg (90%) of pure **1c** as a yellow solid. m.p.: 86–88 °C. ^1^H-NMR (300 MHz, CDCl_3_) δ 8.07 (dd, *J* = 8.0, 1.4 Hz, 1H), 7.16–7.11 (m, 2H), 3.25–3.23 (m, 2H), 2.99–2.96 (m, 2H). ^13^C-NMR (75 MHz, CDCl_3_) δ 193.5, 160.9 (d, *J_C–F_* = 245 Hz), 137.8, 132.7, 129.8 (d, *J_C–F_* = 7 Hz), 121.70 (d, *J_C–F_* = 23.0 Hz), 115.6 (d, *J_C–F_* = 22.6 Hz), 39.7, 27.1. IR ν: 1659, 1595, 1565. HRMS (ESI) calculated for C_9_H_6_FOS [M − H]^−^ 181.0123, found 181.0165.

*2,3,8,9-Tetrahydro-4H,10H-thiopyran[2,3-f]thiochromene-4,10-dione* (**1d**). To a mixture of acrylic acid (288 mg, 280 μL, 4 mmol) and 1,3-benzenedithiol (350 μL, 3 mmol) was added I_2_ (240 mg, 1.0 mmol) and the mixture was stirred at 50 °C for 24 h. After completion of reaction (monitored by TLC), a cold saturated sodium thiosulfate solution (30 mL) was added and extracted with dichloromethane (2 × 30 mL); the combined organic layers were mixed with a saturated solution of sodium bicarbonate to form the diacid salt and extracted to remove the unreacted starting material. The water layer was acidified with hydrochloric acid (10% aq) and extracted with dichloromethane (3 × 50 mL). The combined organic layers were dried over Na_2_SO_4_, evaporation of the solvent under reduced pressure afforded 490 mg (57%); the desired addition product, 100 mg (0.35 mmol) of the later product was cooled down to 0 °C in an ice bath and 3 mL of concentrated sulfuric acid was added, the reaction mixture was allowed to warm to room temperature for 2 h with magnetic stirring and then heated to 60 °C. The reaction was quenched with ice and the mixture was extracted with dichloromethane (3 × 50 mL). The combined organic layers were washed once with water, followed by saturated NaHCO_3_ solution. The combined organic layers were dried over Na_2_SO_4_, and concentrated under reduced pressure. The residue was purified by column chromatography over silica gel using hexane:EtOAc (4:1) as eluent to give 22 mg of **1d**, 25% of pure **2** as a white solid. m.p.: 145–147 °C. ^1^H-NMR (300 MHz, CDCl_3_) δ 8.15 (d, *J* = 8.0 Hz, 1H), 7.08 (d, *J* = 8.0 Hz, 1H), 3.29–3.25 (m, 2H), 3.10–3.05 (m, 4H), 2.99–2.94 (m, 2H). ^13^C-NMR (75 MHz, CDCl_3_) δ 194.3, 193.6, 151.4, 150.1, 132.7, 129.4, 127.6, 123.5, 40.3, 38.7, 27.3, 26.1. IR ν: 1648, 1559, 1351, 834. HRMS (ESI) calculated for C_12_H_11_O_2_S_2_ [M + H]^+^ 251.0200 found 251.0209.

*4-Oxothiochroman-2-carboxylic acid* (**1e**). Thiophenol (110 μL, 1.0 mmol) and furan-2,5-dione (maleic anhydride) (98 mg, 1.0 mmol) were mixed with triethylamine (10 μL). After stirring at 50 °C for 24 h, the mixture was cooled with an ice-cooling bath. AlCl_3_ (approx. 400 mg, 3.0 mmol) was added and the mixture was allowed to warm to room temperature and stirred for 1.5 h. The reaction mixture was quenched with 50 mL of 2.0 M HCl and then extracted with chloroform (4 × 50 mL). The combined organic layers were dried over Na_2_SO_4_, and concentrated under reduced pressure. The residue was purified by column chromatography over silica gel using hexane:EtOAc (1:4) as eluent to give 45 mg (22%) of **1e** as a white solid. m.p.: 151–152 °C. ^1^H-NMR (300 MHz, CDCl_3_) δ 8.09 (d, *J* = 7.8 Hz, 1H), 7.4–7.38 (m, 1H), 7.25–7.21 (m, 2H) 4.13–4.11 (m, 1H), 3.19–3.17 (m, 2H). ^13^C-NMR (75 MHz, CDCl_3_) δ 192.4, 175.2, 138.4, 134.3, 130.6, 129.4, 127.7, 126.3, 42.2, 41.2. IR ν: 3300-2500, 2918, 1680, 1581, 7673. HRMS (ESI) calculated for C_10_H_7_O_3_S [M − H]^−^ 207.0106 found 207.0107.

*6-Nitrothiochroman-4-one* (**1f**). Acrylic acid (510 μL, 504 mg, 7.0 mmol) and 4-nitrothiophenol (1240 mg, 8 mmol) were mixed with 75% aqueous solution of TBAF (512 μL, 1.4 mmol) and the mixture was stirred for 4h at 60 °C. A saturated solution of sodium bicarbonate was added and extracted with dichloromethane (3 × 25 mL) to remove the unreacted starting material. The water layer was acidified with hydrochloric acid (10% aq) and extracted with dichloromethane (3 × 30 mL). The combined organic layers were dried over Na_2_SO_4_, evaporation of the solvent under reduced pressure gave the crude addition product which was dissolved in anhydrous dichloromethane in an oven dried round bottomed flask under N_2_ in an ice cooling bath, oxalyl chloride (850 μL, 7.0 mmol) was added dropwise followed by two drops of DMF and the reaction mixture was allowed to warm to room temperature. After stirring 2.5 h, the solution was cooled to –10 °C, and a solution of 1M SnCl_4_ (8.4 mL, 8.4 mmol) in CH_2_Cl_2_ was added dropwise. The resulting mixture was stirred at 0 °C for 10 min and then allowed to warm to room temperature. After stirring at room temperature for 12 h, water (25 mL) was added and extracted with dichloromethane (3 × 25 mL). The combined organic layers dried over anhydrous Na_2_SO_4_, and concentrated under reduce pressure. The residue was purified by column chromatography over silica gel using hexane:EtOAc (2:1) as eluent to give 1.0 g (68%) of **1f** as a white solid. m.p.: 153–154 °C. ^1^H-NMR (300 MHz, CDCl_3_) δ 8.91 (d, *J* = 2.6 Hz, 1H), 8.18 (dd, *J* = 8.8, 2.6 Hz, 1H), 7.43 (d, *J* = 8.8 Hz, 1H), 3.34 (t, *J* = 6.6 Hz, 2H), 3.04 (t, *J* = 6.6 Hz, 2H). ^13^C-NMR (75 MHz, CDCl_3_) δ 191.7, 150.6, 145.2, 130.8, 128.6, 126.8, 124.4, 38.4, 26.3. IR ν: 1921, 1676, 1594, 1505. HRMS (ESI) calculated for C_9_H_6_NO_3_S [M − H]^−^ 208.0068 found 208.0081.

*6-Aminothiochroman-4-one* (**1g**). To a solution of the 6-nitrothiochroman-4-one **1f**, (170 mg, 0.8 mmol) in 3:1 EtOH/H_2_O (3 mL) was added and iron wire (400 g, 7.3 mmol) and NH_4_Cl (100 mg, 1.8 mmol). The reaction mixture was stirred at 70 °C for 1 h. The mixture was filtered through a small pad of silica gel and then washed with EtOAc. The filtrate was concentrated and the resulting material was dissolved in (10% aq) hydrochloric acid and extracted with dichloromethane (3 × 25 mL) to remove unreacted staring material. The aqueous layer was basified with 2 M NaOH solution and extracted with dichloromethane (3 × 30 mL). The combined organic layers were dried over Na_2_SO_4_, evaporation of the solvent under reduced pressure. The resulting residue was purified by silica gel column chromatography using hexane:EtOAc (3:1) to provide 130 mg (75%) of **1g** as a yellow solid. m.p.: 118–120 °C. ^1^H-NMR (300 MHz, CDCl_3_) δ 7.41 (d, *J* = 2.3 Hz, 1H), 7.06 (d, *J* = 8.4 Hz, 1H), 6.83 (dd, *J* = 8.4, 2.3 Hz, 1H), 3.77 (s, 2H), 3.20–3.06 (m, 2H), 2.95–2.81 (m, 2H). ^13^C-NMR (75 MHz, CDCl_3_) δ 195.3, 143.2, 131.5, 131.4, 128.7, 122.2, 114.9, 39.9, 26.8. IR ν: 3346, 3214, 1887, 1650, 1595. HRMS (ESI) calculated for C_9_H_10_NOS [M + H]^+^ 180.0483 found 180.0485.

#### 4.1.3. Synthesis of Thioflavanones

##### General Procedure I. Horner-Wadsworth-Emmons Reaction. Synthesis of Cinnamic Acids [30]

**Scheme 2:**
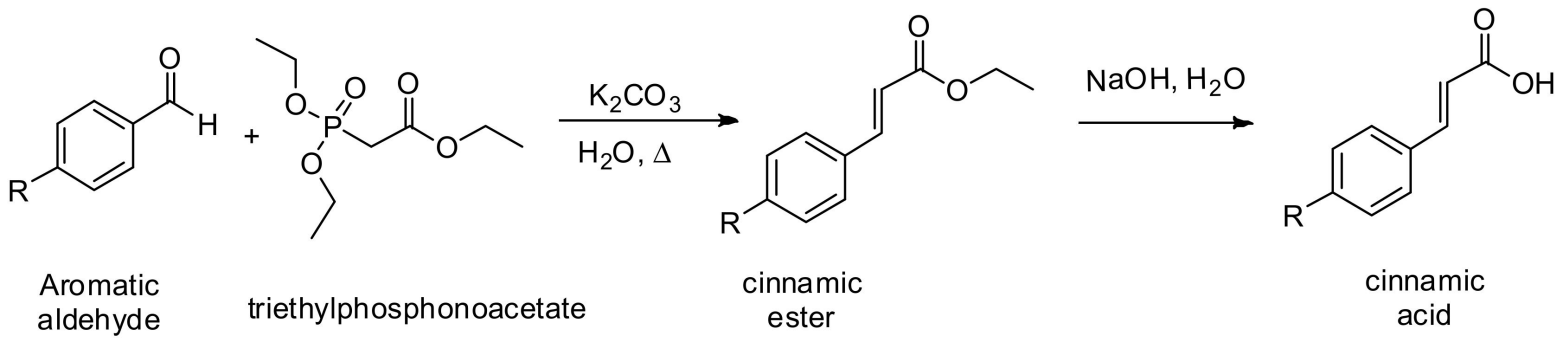
Preparation of cinnamic acid derivatives.

Cinnamic acids were prepared by the hydrolysis of the corresponding ethyl esters which were prepared by Horner-Wadsworth-Emmons reaction between aromatic aldehydes and triethylphosphonoacetate (Scheme 2).

In a round bottomed flask equipped with a reflux condenser and an stirrer were mixed 12.0 mmol of the benzaldehyde, potassium carbonate, K_2_CO_3_ (32 mmol, 2.6 equiv.), 6.0 mL of triethylphosphonoacetate (30 mmol, 2.5 equiv.) and 2.0 mL of water and the reaction mixture was refluxed at 110 °C for 30 min. After completion of the reaction, it was quenched with water and, extracted with dichloromethane (3 × 50 mL). The combined organic layers were dried over Na_2_SO_4_ and concentrated under vacuum. The pure α,β-unsaturated ester was purified by column chromatography and then mixed with a 10% NaOH solution; then, reaction mixture was heated at 80 °C until the completion of the reaction and then 2.0 M HCl solution was added until acid pH, and extracted with chloroform (3 × 50 mL). Combined organic layer were dried over Na_2_SO_4_, and concentrated at reduced pressure, the α,β-unsaturated acid (cinnamic acid) obtained was used without further purification.

##### General Procedure II. Preparation of Thioflavanones

A cinnamic acid derivative (2 mmol) and thiophenol (3 mmol) were mixed with 75% aqueous solution of TBAF (140 μL sln, 0.4 mmol) and the mixture was stirred for 4 h at 60 °C. A saturated solution of sodium bicarbonate was added and extracted with dichloromethane (3 × 25 mL) to remove the unreacted starting material. The water layer was acidified with hydrochloric acid (10% aq) and extracted with dichloromethane (3 × 30 mL). The combined organic layers were dried over Na_2_SO_4_; evaporation of the solvent under reduced pressure gave the crude addition product which was dissolved in anhydrous dichloromethane and placed in an oven-dried round bottomed flask under N_2_ in an ice cooling bath. Consequently, oxalyl chloride (365 μL, 3.0 mmol) was added dropwise followed by two drops of DMF and the reaction mixture is left to warm to room temperature. After stirring for 2.5 h, the solution was cooled to −10 °C, and a solution of 1M SnCl_4_ (3.0 mL, 3.0 mmol) in CH_2_Cl_2_ was added dropwise. The resulting mixture was stirred at 0 °C for 10 min and then allowed to warm to room temperature. After stirring at room temperature for 12 h, water (25 mL) was added and extracted with dichloromethane (3 × 25 mL). The combined organic layers dried over anhydrous Na_2_SO_4_, and concentrated under reduced pressure. The residue was purified by column chromatography over silica gel using hexane:EtOAc (2:1) as eluent to give the desired thioflavanone.

*2-Phenylthiochroman-4-one (thioflavanone)* (**1h**). The title compound was prepared from thiophenol (5.0 mmol) and cinnamic acid (310 mg, 3.0 mmol) according to the general procedure II. Yield 215 mg (45%) white solid. m.p.: 155–157 °C. ^1^H-NMR (300 MHz, CDCl_3_) δ 8.20 (dd, *J* = 7.9, 1.2 Hz, 1H), 7.52–7.36 (m, 6H), 7.35–7.30 (m, 1H), 7.30–7.21 (m, 1H), 4.77 (dd, *J* = 12.7, 3.3 Hz, 1H), 3.56–3.07 (m, 2H). IR ν: 1665, 1586, 1556, 1452, 1433. HRMS (ESI) calculated for C_15_H_13_OS [M + H]^+^ 241.0687, found 241.0694.

*2-(4-(Trifluoromethyl)-phenyl)-thiochroman-4-one* (**1i**). Trifluoromethylcinnamic acid (1070 mg, 5.0 mmol) and thiophenol (660 mg, 6.0 mmol) were mixed with 75% aqueous solution of TBAF (140 μL, 0.4 mmol) and the mixture was stirred for 24 h at 50 °C. A saturated solution of sodium bicarbonate was added and extracted with dichloromethane (3 × 25 mL) to remove the unreacted starting material. The water layer was acidified with hydrochloric acid (10% aq) and extracted with dichloromethane (3 × 50 mL). The combined organic layers were dried over Na_2_SO_4_, evaporation of the solvent under reduced pressure gave the crude addition product which was dissolved in methanesulfonic acid and heated at 60 °C for 2 h. Ice water was added to the mixture and the white precipitate was filtered off and washed with water. The residue was purified by column chromatography over silica gel using hexane:EtOAc (4:1) as eluent to give 650 mg (42%) of **1i** as a white solid. m.p.: 108–110 °C. ^1^H-NMR (300 MHz, CDCl_3_) δ 8.18 (dd, *J* = 7.9, 1.4 Hz, 1H), 7.67 (d, *J* = 8.4 Hz, 2H), 7.58 (d, *J* = 8.4 Hz, 2H), 7.46 (ddd, *J* = 8.0, 7.3, 1.5 Hz, 1H), 7.36–7.22 (m, 3H), 4.79 (dd, *J* = 12.4, 3.3 Hz, 1H), 3.30 (ddd, *J* = 19.7, 16.4, 7.8 Hz, 2H). ^13^C-NMR (75 MHz, CDCl_3_) δ 193.7, 142.4, 141.3, 133.9, 130.8 (q, *J* = 32.4 Hz), 130.5, 129.3, 127.9, 127.3, 126.2 (q, *J* = 3.7 Hz), 125.6, 124.0 (q, *J* = 270.5 Hz), 46.3, 44.9. IR ν: 1677, 1323, 1106, 841, 757. HRMS (ESI) calculated for C_16_H_12_F_3_OS [M + H]^+^ 309.0561, found 309.0572.

*6-Fluoro-2-(4-(trifluoromethyl)-phenyl)thiochroman-4-one* (**1j**). The title compound was prepared from 4-fluorothiophenol (400 μL, 3.0 mmol) and trifluoromethylcinnamic acid (420 mg, 2.0 mmol) according to the general procedure II. Yield 346 mg (53%) of **1j** as a white solid. m.p.: 55–57 °C. ^1^H-NMR (300 MHz, CDCl_3_) δ 7.82 (dd, *J* = 9.2, 2.8 Hz, 1H), 7.63 (d, *J* = 8.2 Hz, 2H), 7.53 (d, *J* = 8.2 Hz, 2H), 7.26 (dd, *J* = 8.9, 5.1 Hz, 1H), 7.17 (ddd, *J* = 8.7, 7.6, 2.9 Hz, 1H), 4.73 (dd, *J* = 11.6, 4.0 Hz, 1H), 3.37–3.15 (m, 2H). ^13^CNMR (75 MHz, CDCl_3_) δ 192.9 (d, *J* = 1.7 Hz), 161.0 (d, *J* = 247.2 Hz), 142.3, 136.6 (d, *J* = 3.0 Hz), 132.0 (d, *J* = 5.9 Hz), 131.0 (q, *J* = 32.7 Hz), 129.3 (d, *J* = 7.0 Hz), 128.1, 126.3 (q, *J* = 3.7 Hz), 124.0 (q, *J* = 272.7 Hz), 122.0 (d, *J* = 23.1 Hz), 115.6 (d, *J* = 22.8 Hz), 46.2, 45.3. IR ν: 1679, 1465, 1404, 1321, 1168, 1109. HRMS (ESI) calculated for C_16_H_11_F_4_OS [M + H]^+^ 327.0467, found 327.0478.

*2-(4-Chlorophenyl)-thiochroman-4-one* (**1k**). The title compound was prepared from thiophenol (440 μL, 4.0 mmol) and 4-chlorocinnamic acid (546 mg, 3.0 mmol) according to the general procedure II. Yield 537 mg (53%) of **1k** as a yellowish solid. m.p.: 123–125 °C. ^1^H-NMR (300 MHz, CDCl_3_) δ 8.12 (dd, *J* = 7.9, 1.3 Hz, 1H), 7.41 (ddd, *J* = 8.1, 7.1, 1.6 Hz, 1H), 7.36–7.30 (m, 4H), 7.29–7.23 (m, 1H), 7.20 (ddd, *J* = 8.2, 7.1, 1.2 Hz, 1H), 4.67 (dd, *J* = 12.2, 3.6 Hz, 1H), 3.21 (m, 2H).^13^C-NMR (75 MHz, CDCl_3_) δ 194.0, 141.6, 137.0, 134.3, 133.8, 130.4, 129.3, 129.2, 128.8, 127.3, 125.5, 46.6, 44.8. IR ν: 1674, 1278, 1082, 851, 767. HRMS (ESI) calculated for C_15_H_10_ClOS [M − H]^−^ 273.0141 found 273.0129.

*2-(4-Fluorophenyl)-thiochroman-4-one* (**1l**) The title compound was prepared from thiophenol (440 μL, 4.0 mmol) and 4-fluorocinnamic acid (500 mg, 3.0 mmol) according to the general procedure II. Yield 485 mg (63%) of **1l** as a yellowish solid. m.p.: 100–102 °C. ^1^H-NMR (300 MHz, CDCl_3_) δ 8.19 (dd, *J* = 8.0, 1.4 Hz, 1H), 7.53–7.39 (m, 3H), 7.29 (ddd, *J* = 15.1, 8.9, 4.6 Hz, 2H), 7.18–7.06 (m, 2H), 4.75 (dd, *J* = 12.4, 3.5 Hz, 1H), 3.28 (qd, *J* = 16.4, 8.0 Hz, 2H). ^13^C-NMR (75 MHz, CDCl_3_) δ 194.2, 162.6 (d, *J* = 247.8 Hz), 141.8, 134.3 (d, *J* = 3.2 Hz), 133.8, 130.4, 129.3, 129.2 (d, *J* = 8.3 Hz), 127.2, 125.4, 116.0 (d, *J* = 21.7 Hz), 46.8, 44.74. IR ν: 2940, 1664, 1582, 1506, 1223, 842, 764. HRMS (ESI) calculated for C_15_H_12_FOS [M + H]^+^ 259.0593, found 259.0598.

*2-(4-Nitrophenyl)-thiochroman-4-one* (**1m**). The title compound was prepared from thiophenol (165 μL, 1.5 mmol) and 4-nitrocinnamic acid (192 mg, 1.0 mmol) according to the general procedure II. Yield 199 mg (70%) of **1m** as a yellowish solid. m.p.: 158–160 °C. ^1^H-NMR (300 MHz, CDCl_3_) δ 8.29 (d, *J* = 8.7 Hz, 2H), 8.20 (dd, *J* = 7.9, 1.2 Hz, 1H), 7.66 (d, *J* = 8.7 Hz, 2H), 7.58–7.43 (m, 1H), 7.34 (d, *J* = 8.7 Hz, 1H), 7.29 (dd, *J* = 9.1, 1.9 Hz, 1H), 4.86 (dd, *J* = 11.3, 3.9 Hz, 1H), 3.46–3.24 (m, 2H). ^13^C-NMR (75 MHz, CDCl_3_) δ 193.2, 147.8, 145.6, 140.7, 134.1, 130.3, 129.4, 128.6, 127.3, 125.8, 124.3, 46.1, 44.7. IR ν: 2920, 1666, 1587, 1514, 1515, 1338, 761. HRMS (ESI) calculated for C_15_H_12_NO_3_S [M + H]^+^ 286.0538, found 286.0543.

#### 4.1.4. General Procedure III. Dehydrogenation with DDQ [22]

A mixture of substrate (0.5 mmol of thiochromanones or thioflavanones), and DDQ (140 mg, 0.6 mmol) and a small amount of anhydrous *p*-toluenesulfonic acid in 2.0 mL of anhydrous benzene, was refluxed in a condenser adapted with a drying tube filled with calcium chloride; after stirring for 24 h (or until the TLC showed the disappearance of the starting materials) the mixture was cooled and the residue was purified by column chromatography using mixtures of ethyl acetate and hexane to give the desired dehydrogenation products in moderate yields.

#### 4.1.5. General Procedure IV. Dehydrogenation with Iodine/DMSO [22]

A mixture of 0.5 mmol of substrate (thiochromanones or thioflavanones) in 2.0 mL of DMSO was added 125 μL of 0.1 M solution of iodine in (25% mol) was refluxed during 24 h, then cooled and poured into water. The mixture was extracted with dichloromethane (3 × 25 mL), and the combined organic layers were dried over sodium sulfate, and then concentrated at reduced pressure. The residue was purified in column chromatography using mixtures of ethyl acetate and hexane to give the desired dehydrogenation products in moderate yields.

*6-Nitro-4H-thiochromen-4-one* (**2f**). The title compound was prepared from **1f** according to the general procedure III. Yield 50 mg (48%) of **2f** as a yellowish solid. mp 184–185 °C. ^1^H-NMR (300 MHz, CDCl_3_) δ 9.32 (d, *J* = 2.5 Hz, 1H), 8.39 (dd, *J* = 8.9, 2.5 Hz, 1H), 7.85 (d, *J* = 10.5 Hz, 1H), 7.77 (d, *J* = 8.9 Hz, 1H), 7.06 (d, *J* = 10.5 Hz, 1H). ^13^C-NMR (75 MHz, CDCl_3_) δ 178.5, 147.4, 144.0, 137.9, 132.9, 128.5, 126.6, 125.4, 124.6. IR ν: 3096, 3031, 1602, 1505, 1339. HRMS (ESI) calculated for C_9_H_6_NO_3_S [M + H]^+^ 208.0068 found 208.0077.

*2-(4-(Trifluoromethyl)-phenyl)-4H-thiochromen-4-one* (**2i**). The title compound was prepared from **1i** according to the general procedure III. Yield 69 mg (45%) of **21** as a yellowish solid. m.p.: 168–170 °C. ^1^H-NMR (300 MHz, CDCl_3_) δ 8.54 (d, *J* = 7.6 Hz, 1H), 7.85–7.71 (m, 4H), 7.69–7.51 (m, 3H), 7.24 (s, 1H). ^13^C-NMR (75 MHz, CDCl_3_) δ 180.6, 151.2, 140.0, 137.3, 132.6 (d, *J* = 32.9 Hz) *, 131.9, 130.8, 128.7, 128.1, 127.5, 126.5, 126.3 (q, *J* = 3.7 Hz), 124.4, 123.6 (d, *J* = 272.7 Hz). IR ν: 3033, 2930, 1615, 1438, 1113, 667. HRMS (ESI) calculated for C_16_H_10_F_3_OS [M + H]^+^ 307.0404 found 307.0446.

*6-Fluoro-2-(4-(trifluoromethyl)-phenyl)-4H-thiochromen-4-one* (**2j**). The title compound was prepared from **1j** according to the general procedure III. Yield 90 mg (55%) of **2j** as a yellowish solid. m.p.: 128–130 °C. ^1^H-NMR (300 MHz, CDCl_3_) δ 8.20 (dd, *J* = 9.3, 2.8 Hz, 1H), 7.82–7.72 (m, 4H), 7.67 (dd, *J* = 8.8, 4.7 Hz, 1H), 7.41 (ddd, *J* = 8.8, 7.6, 2.9 Hz, 1H), 7.22 (s, 1H). ^13^C-NMR (75 MHz, CDCl_3_) δ 180.0 (d, *J* = 2.2 Hz), 162.6 (d, *J* = 250.4 Hz), 151.8, 140.0, 133.1 (d, *J* = 7.5 Hz), 133.0 (q, *J* = 33.2 Hz), 132.8 (d, *J* = 3.7 Hz), 128.9 (d, *J* = 7.7 Hz), 127.7, 126.6 (q, *J* = 3.7 Hz), 123.8 (q, *J* = 272.4 Hz), 123.7, 121.0 (d, *J* = 24.2 Hz), 114.6 (d, *J* = 23.0 Hz). IR ν: 2923, 1713, 1623, 1605, 1118. HRMS (ESI) calculated for C_16_H_9_F_4_OS [M + H]^+^ 325.0310 found 325.0331.

*2-(4-Chlorophenyl)-4H-thiochromen-4-one* (**2k**). The title compound was prepared from **1k** according to the general procedure III. Yield 82 mg (60%) of **2k** as a yellowish solid. m.p.: 161–162 °C. ^1^H-NMR (300 MHz, CDCl_3_) δ 8.60–8.49 (m, 1H), 7.74–7.53 (m, 5H), 7.52–7.42 (m, 2H), 7.37 (s, 1H). ^13^C-NMR (75 MHz, CDCl_3_) δ 181.0, 151.9, 137.6, 137.4, 135.2, 132.0, 131.0, 129.8, 128.9, 128.4, 128.2, 126.7, 123.8. IR ν: 3014, 1627, 1588, 1087, 777. HRMS (ESI) calculated for C_15_H_10_ClOS [M + H]^+^ 273.0141 found 273.0172.

*2-(4-Fluorophenyl)-4H-thiochromen-4-one* (**2l**). The title compound was prepared from **1l** according to the general procedure III. Yield 74 mg (58%) of **2l** as a yellowish solid. m.p.: 160–161 °C. ^1^H-NMR (300 MHz, CDCl_3_) δ 8.62 (d, *J* = 7.7 Hz, 1H), 7.82–7.69 (m, 4H), 7.68–7.60 (m, 1H), 7.47 (s, 1H), 7.26 (t, *J* = 8.5 Hz, 2H). ^13^C-NMR (75 MHz, CDCl_3_) δ 180.8, 164.4 (d, *J* = 252.0 Hz), 151.9, 137.5, 132.8 (d, *J* = 3.3 Hz), 131.8, 130.8, 129.0 (d, *J* = 8.6 Hz), 128.7, 127.9, 126.5, 123.5, 116.5 (d, *J* = 22.1 Hz). IR ν: 2921, 1608, 1582, 1500, 1225, 845. HRMS (ESI) calculated for C_15_H_10_FOS [M + H]^+^ 257.0436 found 257.0463.

*2-(4-nitrophenyl)-4H-thiochromen-4-one* (**2m**). The title compound was prepared from **1m** according to the general procedure IV. Yield 128 mg (91%) of **2m** as a yellowish solid. mp 183–185 °C. ^1^H-NMR (300 MHz, CDCl_3_) δ 8.54 (d, *J* = 7.8 Hz, 1H), 8.35 (d, *J* = 8.7 Hz, 2H), 7.85 (d, *J* = 8.7 Hz, 2H), 7.72–7.62 (m, *J* = 3.0 Hz, 2H), 7.62–7.53 (m, 1H), 7.25 (s, 1H). ^13^C-NMR (75 MHz, CDCl_3_) δ 180.7, 150.3, 149.3, 142.7, 137.2, 132.3, 131.0, 129.0, 128.5, 128.3, 126.8, 125.2, 124.7. IR ν: 3015, 1738, 1628, 1513, 1354, 850. HRMS (ESI) calculated for C_15_H_10_NO_3_S [M + H]^+^ 284.0381 found 284.0407.

#### 4.1.6. General Procedure V. Oxidation of Sulfides to Sulfones.

To a 25-mL glass tube, sulfide (1.0 mmol), oxone (0.9221 g, 1.5 mmol), and a water ethanol mixture (1:1) (3.0 mL) were added and the mixture was stirred at 60 °C for 12 h. The mixture was then cooled to room temperature and extracted with dichloromethane (3 × 25 mL). The combined organic layers were dried over anhydrous Na_2_SO_4_ and concentrated under reduced pressure. The residue was purified by column chromatography over silica gel using mixtures of hexane and EtOAc as eluent.

*2-Methylthiochroman-4-one 1,1-dioxide* (**3a**). The title compound was prepared from **1a** according to the general procedure V. Yield 178 mg (82%) of **3a** as a white solid. m.p.: 129–131 °C. ^1^H-NMR (300 MHz, CDCl_3_) δ 8.10 (dd, *J* = 7.7, 1.0 Hz, 1H), 8.04 (dd, *J* = 7.7, 0.8 Hz, 1H), 7.81 (td, *J* = 7.6, 1.3 Hz, 1H), 7.72 (td, *J* = 7.6, 1.2 Hz, 1H), 3.76 (dd, *J* = 14.2, 6.9 Hz, 1H), 3.33–3.19 (m, 2H), 1.54 (d, *J* = 6.9 Hz, 3H). ^13^C-NMR (75 MHz, CDCl_3_) δ 190.4, 140.7, 135.0, 133.3, 130.5, 128.5, 124.2, 54.6, 44.2, 11.7. IR ν: 3082, 2945, 1694, 1312, 1151, 728. HRMS (ESI) calculated for C_10_H_9_O_3_S [M − H]^−^ 209.0272 found 209.0282.

*6-Fluoro-2-methylthiochroman-4-one 1,1-dioxide* (**3b**). The title compound was prepared from **1b** according to the general procedure V. Yield 161 mg (71%) of **3b** as a white solid. m.p.: 119–120 °C. ^1^H-NMR (300 MHz, CDCl_3_) δ 8.06 (dd, *J* = 8.7, 4.9 Hz, 1H), 7.75 (dd, *J* = 8.7, 2.6 Hz, 1H), 7.55–7.42 (m, 1H), 3.84–3.62 (m, 1H), 3.37–3.15 (m, 2H), 1.53 (2, *J* = 10.5 Hz, 3H). ^13^C-NMR (75 MHz, CDCl_3_) δ 189.4, 165.2 (d, *J* = 257.5 Hz), 136.9 (d, *J* = 3.6 Hz), 133.4 (d, *J* = 7.2 Hz), 127.5 (d, *J* = 8.6 Hz), 122.4 (d, *J* = 23.0 Hz), 115.4 (d, *J* = 23.8 Hz), 54.7, 44.3, 11.7. IR ν: 3100, 2939, 1697, 1580, 1310, 1273, 1147, 694. HRMS (ESI) calculated for C_10_H_8_FO_3_S [M − H]^−^ 227.0178 found 227.0179.

*6-Fluorothiochroman-4-one 1,1-dioxide* (**3c**). The title compound was prepared from **1c** according to the general procedure V. Yield 174 mg (81%) of **3c** as a white solid. m.p.: 175–177 °C. ^1^H-NMR (300 MHz, CDCl_3_) δ 8.03 (dd, *J* = 8.7, 4.9 Hz, 1H), 7.76 (dd, *J* = 8.7, 2.6 Hz, 1H), 7.48 (ddd, *J* = 8.7, 7.7, 2.7 Hz, 1H), 3.74–3.62 (m, 2H), 3.48–3.37 (m, 2H). ^13^C-NMR (75 MHz, CDCl_3_) δ 189.1, 165.2 (d, *J* = 257.6 Hz), 137.6 (d, *J* = 3.6 Hz), 133.1 (d, *J* = 7.3 Hz), 126.9 (d, *J* = 8.7 Hz), 122.3 (d, *J* = 23.0 Hz), 115.7 (d, *J* = 23.8 Hz), 49.3, 36.9. IR ν: 3087, 2980, 1690, 1577, 1271, 1130, 749. HRMS (ESI) calculated for C_9_H_6_FO_3_S [M − H]^−^ 213.0022 found 213.0025.

*2-Phenylthiochroman-4-one 1,1-dioxide* (**3h**). The title compound was prepared from **1h** according to the general procedure V. Yield 217 mg (80%) of **3h** as a white solid. m.p.: 154–155 °C. ^1^H-NMR (300 MHz, CDCl_3_) δ 8.22–8.12 (m, 1H), 8.07 (d, *J* = 7.4 Hz, 1H), 7.90–7.72 (m, 2H), 7.61–7.35 (m, 5H), 4.86 (dd, *J* = 12.8, 3.2 Hz, 1H), 3.96 (dd, *J* = 17.7, 12.8 Hz, 1H), 3.41 (dd, *J* = 17.7, 3.2 Hz, 1H). ^13^C-NMR (75 MHz, CDCl_3_) δ 190.9, 141.5, 135.1, 133.4, 130.5, 130.1, 129.9, 129.2, 128.8, 128.0, 124.5, 64.1, 43.1. IR ν: 2957, 1693, 1586, 1281, 1149, 1120, 764. HRMS (ESI) calculated for C_15_H_11_O_3_S [M − H]^−^ 271.0429 found 271.0428.

*2-(4-(Trifluoromethyl)-phenyl)-thiochroman-4-one 1,1-dioxide* (**3i**). The title compound was prepared from **1i** according to the general procedure V. Yield 146 mg (43%) of **3i** as a white solid. m.p.: 155–157 °C. ^1^H-NMR (300 MHz, CDCl_3_) δ 8.18 (dd, *J* = 7.7, 1.2 Hz, 1H), 8.07 (dd, *J* = 7.7, 1.0 Hz, 1H), 7.85 (td, *J* = 7.6, 1.5 Hz, 1H), 7.78 (td, *J* = 7.6, 1.3 Hz, 1H), 7.72 (d, *J* = 8.2 Hz, 2H), 7.61 (d, *J* = 8.3 Hz, 2H), 4.93 (dd, *J* = 12.8, 3.2 Hz, 1H), 3.96 (dd, *J* = 17.7, 12.8 Hz, 1H), 3.41 (dd, *J* = 17.7, 3.3 Hz, 1H). ^13^C-NMR (75 MHz, CDCl_3_) δ 190.4, 141.3, 135.5, 133.87, 132.4 (q, *J* = 33Hz), 132.1, 130.6, 130.5, 129.1, 126.3 (dd, *J* = 7.5, 3.7 Hz), 124.7, 123.9 (q, *J* = 272.5 Hz), 63.8, 43.0. IR ν: 2927, 1691, 1294, 1153, 712. HRMS (ESI) calculated for C_16_H_12_F_3_O_3_S [M + H]^+^ 341.0459 found 341.0465.

*6-Fluoro-2-(4-(trifluoromethyl)-phenyl)-thiochroman-4-one 1,1-dioxide* (**3j**) The title compound was prepared from **1j** according to the general procedure V. Yield 254 mg (62%) of **3j** as a white solid. m.p.: 132–134 °C. ^1^H-NMR (300 MHz, CDCl_3_) δ 8.09 (dd, *J* = 8.7, 4.8 Hz, 1H), 7.83 (dd, *J* = 8.6, 2.6 Hz, 1H), 7.72 (d, *J* = 8.2 Hz, 2H), 7.60 (d, *J* = 8.2 Hz, 2H), 7.56–7.47 (m, 1H), 4.92 (dd, *J* = 12.7, 3.1 Hz, 1H), 3.97 (dd, *J* = 17.8, 12.8 Hz, 1H), 3.44 (dd, *J* = 17.8, 3.2 Hz, 1H). ^13^C-NMR (75 MHz, CDCl_3_) δ 189.1, 165.3 (d, *J* = 258.3 Hz), 137.1 (d, *J* = 3.6 Hz), 133.2 (d, *J* = 7.4 Hz), 132.3 (d, *J* = 33.0 Hz), 131.6, 130.3, 127.7 (d, *J* = 8.7 Hz), 126.1 (q, *J* = 3.5 Hz), 123.6 (q, *J* = 272.4 Hz), 122.5 (d, *J* = 23.0 Hz), 115.7 (d, *J* = 23.9 Hz), 63.6, 42.9. IR ν: 2918, 1697, 1579, 1298, 1158, 1114. HRMS (ESI) calculated for C_16_H_9_F_4_O_3_S [M − H]^−^ 357.0209 found 357.0201.

*2-(4-Chlorophenyl)-thiochroman-4-one 1,1-dioxide* (**3k**). The title compound was prepared from **1k** according to the general procedure V. Yield 199 mg (65%) of **3k** as a white solid. m.p.: 160–161 °C. ^1^H-NMR (300 MHz, CDCl_3_) δ 8.16 (dd, *J* = 7.6, 1.1 Hz, 1H), 8.05 (dd, *J* = 7.7, 0.8 Hz, 1H), 7.83 (td, *J* = 7.6, 1.4 Hz, 1H), 7.76 (td, *J* = 7.6, 1.2 Hz, 1H), 7.47–7.36 (m, 4H), 4.83 (dd, *J* = 12.8, 3.2 Hz, 1H), 3.90 (dd, *J* = 17.7, 12.8 Hz, 1H), 3.37 (dd, *J* = 17.7, 3.2 Hz, 1H).^13^C-NMR (75 MHz, CDCl_3_) δ 190.7, 141.4, 136.5, 135.4, 133.7, 131.3, 130.6, 129.6, 129.0, 126.6, 124.7, 63.6, 43.1. IR ν: 2932, 1687, 1584, 1490 1314, 1280, 1147. HRMS (ESI) calculated for C_15_H_10_ClO_3_S [M − H]^−^ 305.0039 found 305.0038.

*2-(4-Fluorophenyl)thiochroman-4-one 1,1-dioxide* (**3l**). The title compound was prepared from **1l** according to the general procedure V. Yield 180 mg (62%) of **3l** as a white solid. m.p.: 121–123 °C. ^1^H-NMR (300 MHz, CDCl_3_) δ 8.17 (d, *J* = 7.6 Hz, 1H), 8.06 (d, *J* = 7.8 Hz, 1H), 7.83 (t, *J* = 7.6, 1.2 Hz, 1H), 7.77 (t, *J* = 10.8, 4.3 Hz, 1H), 7.46 (dd, *J* = 8.6, 5.2 Hz, 2H), 7.14 (t, *J* = 8.6 Hz, 2H), 4.85 (dd, *J* = 12.9, 3.1 Hz, 1H), 3.91 (dd, *J* = 17.7, 12.9 Hz, 1H), 3.38 (dd, *J* = 17.7, 3.2 Hz, 1H). ^13^C-NMR (75 MHz, CDCl_3_) δ 190.8, 163.9 (d, *J* = 250.2 Hz), 141.5, 135.4, 133.7, 131.9 (d, *J* = 8.7 Hz), 130.6, 129.0, 124.7, 123.9 (d, *J* = 3.0 Hz), 116.5 (d, *J* = 21.8 Hz), 63.4, 43.3. IR ν: 2927, 1697, 1589, 1506, 1490, 1314, 1284, 1152. HRMS (ESI) calculated for C_15_H_10_FO_3_S [M − H]^−^ 289.0335 found 289.0336.

*2-(4-Nitrophenyl)-thiochroman-4-one 1,1-dioxide* (**3m**). The title compound was prepared from **1m** according to the general procedure V. Yield 237 mg (75%) of **3m** as a white solid. m.p.: 180–182 °C. ^1^H-NMR (300 MHz, CDCl_3_) δ 8.31 (d, *J* = 8.8 Hz, 2H), 8.19 (dd, *J* = 7.6, 1.2 Hz, 1H), 8.06 (dd, *J* = 7.6, 0.9 Hz, 1H), 7.86 (td, *J* = 7.6, 1.5 Hz, 1H), 7.80 (td, *J* = 7.5, 1.3 Hz, 1H), 7.68 (d, *J* = 8.7 Hz, 2H), 4.98 (dd, *J* = 12.8, 3.2 Hz, 1H), 3.97 (dd, *J* = 17.6, 12.8 Hz, 1H), 3.43 (dd, *J* = 17.6, 3.2 Hz, 1H). ^13^CNMR (75 MHz, CDCl_3_) δ 189.9, 149.1, 141.1, 135.5, 135.1, 134.0, 131.2, 130.5, 129.2, 124.7, 124.4, 63.6, 42.9. IR ν: 3078, 2921, 1695, 1586, 1517, 1342, 1155. HRMS (ESI) calculated for C_15_H_10_NO_5_S [M − H]^−^ 316.0280 found 316.0267.

*2-Phenyl-4H-thiochromen-4-one 1,1-dioxide* (**4h**). The title compound was prepared from **3h** according to the general procedure IV. Yield 120 mg (89%) of **4h** as a white solid. m.p.: 134–135 °C. ^1^H-NMR (300 MHz, CDCl_3_) δ 8.21 (dd, *J* = 7.8, 0.7 Hz, 1H), 8.14–8.05 (m, 1H), 7.87 (ddd, *J* = 8.3, 5.5, 1.2 Hz, 3H), 7.76 (t, *J* = 7.3 Hz, 1H), 7.60–7.43 (m, 3H), 6.82 (s, 1H). ^13^C-NMR (75 MHz, CDCl_3_) δ 178.3, 153.1, 141.7, 134.8, 133.0, 131.9, 129.3, 128.8, 128.7 (2CHs), 128.5, 128.1, 123.6. IR ν: 2920, 2849, 1964, 1650, 1588, 1287, 1242, 1149. HRMS (ESI) calculated for C_15_H_11_O_3_S [M + H]^+^ 271.0429 found 271.0434.

*2-(4-(Trifluoromethyl)-phenyl)-4H-thiochromen-4-one 1,1-dioxide* (**4i**). The title compound was prepared from **3i** according to the general procedure IV. Yield 167 mg (99%) of **4i** as a white solid. m.p.: 188–189 °C. ^1^H-NMR (300 MHz, CDCl_3_) δ 8.22 (dd, *J* = 7.9, 1.5 Hz, 1H), 8.10 (dd, *J* = 7.9, 1.4 Hz, 1H), 7.96 (d, *J* = 8.4 Hz, 2H), 7.90 (td, *J* = 7.6, 1.5 Hz, 1H), 7.79 (td, *J* = 7.5, 1.2 Hz, 1H), 7.77 (dd, *J* = 7.7, 1.2 Hz, 2H), 6.82 (s, *J* = 5.8 Hz, 1H). ^13^C-NMR (75 MHz, CDCl_3_) δ 178.2, 152.1, 141.6, 135.3, 133.6 (q, *J* = 33.1), 133.4, 132.4, 130.3, 129.6, 128.6, 128.5, 126.4 (q, *J* = 3.6 Hz), 123.9, 123.7 (q, *J* = 272.8). IR ν: 2917, 1651, 1587, 1295, 1119, 837. HRMS (ESI) calculated for C_16_H_10_F_3_O_3_S [M + H]^+^ 339.0303 found 339.0306.

*6-Fluoro-2-(4-(trifluoromethyl)-phenyl)-4H-thiochromen-4-one 1,1-dioxide* (**4j**). The title compound was prepared from **3j** according to the general procedure IV. Yield 144 mg (81%) of **4j** as a white solid. m.p.: 172–173°C. ^1^H-NMR (300 MHz, CDCl_3_) δ 8.12 (dd, *J* = 8.7, 4.7 Hz, 1H), 7.94 (d, *J* = 8.1 Hz, 2H), 7.86 (dd, *J* = 8.5, 2.6 Hz, 1H), 7.77 (d, *J* = 8.3 Hz, 2H), 7.71–7.46 (m, 1H), 6.83 (s, 1H). ^13^C-NMR (75 MHz, CDCl_3_) δ 177.2, 165.1 (d, *J* = 258.3 Hz), 152.5, 137.7 (d, *J* = 3.8 Hz), 133.9 (q, *J* = 33.3 Hz), 132.1, 131.4 (d, *J* = 7.6 Hz), 129.9, 129.7, 127.1 (d, *J* = 8.8 Hz), 126.5 (q, *J* = 3.6 Hz), 123.7 (q, *J* = 272.6 Hz), 122.9 (d, *J* = 23.3 Hz), 115.3 (d, *J* = 24.0 Hz). IR ν: 3040, 2918, 1658, 1580, 1292, 1119. HRMS (ESI) calculated for C_16_H_9_F_4_O_3_S [M + H]^+^ 357.0209 found 357.0210.

*2-(4-Chlorophenyl)-4H-thiochromen-4-one 1,1-dioxide* (**4k**). The title compound was prepared from **3k** according to the general procedure IV. Yield 144 mg (95%) of **4k** as a white solid. m.p.: 173–174 °C. ^1^H-NMR (300 MHz, CDCl_3_) δ 8.21 (d, *J* = 7.9 Hz, 1H), 8.09 (d, *J* = 7.9 Hz, 1H), 7.88 (t, *J* = 7.7 Hz, 1H), 7.80 (d, *J* = 8.2 Hz, 2H), 7.77 (t, *J* = 7.5 Hz, 1H), 7.49 (d, *J* = 8.2 Hz, 2H), 6.80 (s, 1H).^13^C-NMR (75 MHz, CDCl_3_) δ 178.3, 152.3, 141.7, 138.7, 135.2, 133.3, 130.4, 129.9, 129.2, 128.7, 128.4, 127.3, 123.9. IR ν: 2916, 1644, 1297, 1090. HRMS (ESI) calculated for C_15_H_10_ClO_3_S [M + H]^+^ 305.0039 found 305.0036.

*2-(4-Fluorophenyl)-4H-thiochromen-4-one 1,1-dioxide* (**4l**). The title compound was prepared from **3l** according to the general procedure IV. Yield 36 mg (25%) of **4l** as a white solid. m.p.: 173–174 °C. ^1^H-NMR (300 MHz, CDCl_3_) δ 8.21 (dd, *J* = 7.9, 1.0 Hz, 1H), 8.09 (dd, *J* = 7.9, 0.7 Hz, 1H), 7.94–7.82 (m, 3H), 7.77 (td, *J* = 7.7, 1.1 Hz, 1H), 7.21 (t, *J* = 8.7 Hz, 2H), 6.78 (s, 1H). ^13^C-NMR (75 MHz, CDCl_3_) δ 178.4, 165.2 (d, *J* = 254.3 Hz), 152.3, 141.8, 135.1, 133.3, 131.4 (d, *J* = 8.9 Hz), 129.0, 128.7, 128.4, 125.0 (d, *J* = 3.3 Hz), 123.9, 116.9 (d, *J* = 22.1 Hz). IR ν: 2849, 1650, 1585, 1505, 1286, 1147. HRMS (ESI) calculated for C_15_H_10_FO_3_S [M + H]^+^ 289.0335 found 289.0335.

*2-(4-Nitrophenyl)-4H-thiochromen-4-one 1,1-dioxide* (**4m**). The title compound was prepared from **3m** according to the general procedure IV. Yield 99 mg (63%) of **4m** as a white solid. m.p.: 187–189 °C. ^1^H-NMR (300 MHz, CDCl_3_) δ 8.43 (d, *J* = 8.4 Hz, 2H), 8.30 (d, *J* = 7.8 Hz, 1H), 8.17 (d, *J* = 7.9 Hz, 1H), 8.09 (d, *J* = 8.4 Hz, 2H), 7.99 (t, *J* = 7.7 Hz, 1H), 7.87 (t, *J* = 7.6 Hz, 1H), 6.92 (s, 1H). ^13^C-NMR (75 MHz, CDCl_3_) δ 177.8, 151.2, 149.7, 141.2, 135.3, 134.8, 133.4, 130.6, 130.2, 128.42, 128.38, 124.3, 123.7. IR ν: 2917, 2849, 1651, 1586, 1512, 1286, 1147. HRMS (ESI) calculated for C_15_H_10_NO_5_S [M + H]^+^ 316.0280 found 316.0281.

### 4.2. Biological Activity

#### 4.2.1. Cytotoxic Activity

Cytotoxicity of the compounds was evaluated over human monocytes (U-937 ATCC CRL-1593.2) in exponential growing phase and, adjusted at 1 × 10^5^ cells/mL in RPMI-1640 enriched with 10% fetal bovine serum (FBS). One hundred microliters of cell suspension were dispensed in each well of a 96-wells microplate and then, 100 μL of each compound or standard drug (amphotericin B) at four serial dilution concentrations (200, 50, 12.5 and 3.125 μg/mL) were added dissolved in pbs with 0.5% DMSO. Cell exposed to compounds or standard drugs were incubated 72 h at 37 °C and 5% of CO_2_. Cytotoxic activity of each compound was determined according to the effect on the cell viability by the MTT microenzymatic method in which 3-(4,5-dimethylthiazol-2-yl)- 2,5-diphenyltetrazolium bromide is reduced to a purple product named formazan by mitochondrial enzyme succinate dehydrogenase. Thus, 10 μL/well of MTT solution (5 mg/mL) were added to each well of exposed and unexposed cells, and plates were incubated at 37 °C, 5% CO_2_ during 3 h. The reaction was stopped by adding 100 μL/well of isopropanol with 50% and 10% of SDS (sodium dodecyl sulfate). The concentration of formazan was determined spectrophotometrically at 570 nm (Varioskan Flash Multimode Reader, Thermo Scientific, Waltham, MA, USA) and intensity of color (absorbance) was registered as O.D. Cells exposed to control drug (amphotericin B) were used as control for toxicity (positive control) while cell incubated in absence of any compound or drug were used as control for viability (negative control). Non-specific absorbance was corrected by subtracting absorbance (O.D) of the blank. Determinations were done by triplicate in at least two independent experiments [31].

#### 4.2.2. Antileishmanial Activity

Antileishmanial activity of compounds was determined according to the ability of the compound to reduce the infection by *L. panamensis* parasites. For this, the antileishmanial activity was tested on intracellular amastigotes of *L. panamensis* transfected with the green fluorescent protein gene (MHOM/CO/87/UA140-EGFP strain) [24]. Briefly, U-937 human cells at a density of 3 × 10^5^ cells/mL in RPMI 1640 and 0.1 μg/mL of PMA (phorbol-12-myristate-13-acetate) were dispensed on 24-wells microplate and then infected with stationary phase growing *L. panamensis* promastigotes in 15:1 parasites per cell ratio. Plates were incubated at 34 °C and 5% CO_2_ for 3 h and then cells were washed twice with phosphate buffer solution (PBS) to eliminate not internalized parasites. Fresh RPMI-1640 was added into each well (1 mL) and plates were incubated again. After 24 h of infection, the RPMI-1640 medium was replaced by fresh culture medium containing each compound at four serial dilutions (50, 12.5, 3.125 and 0.78 μg/mL) and plates were then incubated at 37 °C and 5% CO_2_ during 72 h, then, cells were removed from the bottom plate with 100 μL of EDTA/Trypsin (250 mg) solution. The cells were centrifuged at 1100 rpm during 10 min at 4 °C, the supernatant was discarded and cells were washed with 1 mL of cold PBS and centrifuged at 1100 rpm for 10 min at 4 °C. Cells were washed two times employing PBS, as previously, and after the last wash, the supernatant was discarded and cells were suspended in 500 μL of PBS.

Cells were analyzed by flow cytometry employing a flow cytometer (cytomics FC 500MPL, Beckman Coulter. Pasadena, CA, USA) reading at 488 nm (exciting) and 525 nm (emitting) over an argon laser and counting 10,000 events. Infected cells were determined according the events for green fluorescence (parasites). All determinations for each compound and standard drug were carried out by triplicate, in two experiments. Infected cells exposed to control drug (amphotericin B) were used as control for antileishmanial activity (positive control) while infected cells incubated in absence of any compound or drug were used as control for infection (negative control). Nonspecific fluorescence was corrected by subtracting fluorescence of unstained cells. Determinations were done by triplicate in at least two independent experiments [24,32].

#### 4.2.3. Statistical Analysis

Cytotoxicity was determined according to viability and mortality percentages obtained for each experimental condition (synthetized compounds, amphotericin B and culture medium). Results were expressed as the mean lethal concentrations (LC_50_), concentration necessary to kill 50% of cells, calculated by the parametric method of linear regression that permits doses-response analysis (Probit analysis) [32].

Initially, viability percentages were calculated by Equation (1), where the O.D of control well, corresponds to 100% of viability.

% viability = (O.D exposed cells/O.D unexposed cells) × 100(1)

Then, the percentage of cell growth inhibition was calculated using Equation (2):

% inhibition = 100 − (% Viability)(2)

The toxicity was defined according to LC_50_ values, using the follow scale: Toxic; LC_50_ < 100 μM; moderately toxic; LC_50_ > 100 μM and <200 μM and potentially nontoxic; LC_50_ > 200 μM.

Antileishmanial activity was determined according reduction of percentage of fluorescent parasites determined according to the median fluorescence intensity (MFI), obtained for each experimental condition by cytometry. The parasite values for each concentration of compound were calculated by Equation (3), where the % of parasites in the control well, corresponds to 100% of parasites.

% parasites = (MFI exposed parasites/MFI unexposed parasites) × 100(3)

Then, inhibition percentage was calculated with Equation (4):

% inhibition of parasites = 100 − (% parasites)(4)

Results of antileishmanial activities were expressed as the median effective concentrations (EC_50_) measured by Probit method. The activity of each compound was established according to EC_50_ values as: high activity: EC_50_ < 25 μM; moderate activity: EC_50_ >25 μM and <100 μM and low activity: EC_50_ > 100 μM.

## 5. Conclusions

In summary, 34 thiochromone derivatives were synthesized and their leishmanicidal and cytotoxic activities were evaluated; many compounds were found to possess weak to moderate activities against intracellular amastigotes of *L. panamensis*. However, sulfone derivatives bearing an α,β-unsaturated carbonyl moiety (**4h, 4i, 4j, 4k, 4l, 4m**) displayed the higher antileishmanial activity and the highest IS; accordingly, the most potent antileishmanial agent was the compound **4j** (EC_50_ = 3.14 ± 0.23 μM, IS > 173.24), even higher than the reference drug amphotericin B (IS = 132.02).

Electron-withdrawing groups in the *para* position of the phenyl ring do not affect the activity; however, the fluorine atom at C6 increases the antileishmanial activity and selectivity. The sulfone, in addition to the α,β-unsaturated carbonyl groups in the thiochromone moiety, were the most responsible for the antileishmanial activity. This scaffold could be considered a starting point for further optimization.

## Figures and Tables

**Table 1:** In vitro antileishmanial and cytotoxic activities.

Compound	EC_50_ (μM) ^1^	LC_50_ (μM) ^1^	SI ^2^
**1a**	444.60 ± 7.29	604.21 ± 86.40	1.36
**1b**	422 ± 9.17	578.88 ± 59.11	1.37
**1c**	>109.88	705.2	<6.42
**1d**	>80.00	279.22 ± 36.35	<3.49
**1e**	>96.14	142.63 ± 16.80	<1.48
**1f**	>95.59	>200	<10.00
**1g**	>111.58	118.08 ± 10.51	<1.100
**1h**	44.06 ± 0.90	>41.61	<0.94
**1i**	61.43 ± 1.62	> 648.7	>10.56
**1j**	80.29 ± 12.09	128.65 ± 3.45	1.60
**1k**	>72.79	64.36 ± 3.98	<0.90
**1l**	93.57 ± 10.99	72.86 ± 9.87	0.78
**1m**	>70.1	>700.97	~10.00
**2f**	36.76 ± 1.74	>965.20	>26.26
**2i**	160.97 ± 20.53	522.33 ± 314.03	3.24
**2j**	52.26 ± 8.92	51.39 ± 11.55	0.98
**2k**	>73.3	>733.27	~10.00
**2l**	>78.04	503.32 ± 381.94	<6.45
**2m**	>70.6	>706	~10.00
**3a**	>95.22	>825.2	~8.67
**3b**	>87.71	>876.3	~9.99
**3c**	>93.45	>933.6	~9.99
**3h**	217.02 ± 34.89	528.90 ± 52.88	0.41
**3i**	84.81 ± 3.23	62.88 ± 10.28	0.74
**3j**	79.11± 8.98	>558.18	>7.06
**3k**	137.09 ± 1.29	>651.98	>4.76
**3l**	>68.9	688.92	<10.0
**3m**	>63.03	>630.28	~10.0
**4h**	**7.56 ± 0.33**	**>739.92**	**>97.94**
**4i**	**7.30 ± 1.60**	**>591.19**	**>80.97**
**4j**	**3.24 ± 0.23**	**>561.34**	**>173.24**
**4k**	**7.47 ± 0.75**	**>656.28**	**>87.70**
**4l**	**6.72 ± 0.16**	**>693.75**	**>103.26**
**4m**	**7.23 ± 0.44**	**>634.32**	**>87.70**
***Amphotericin B***	**0.32 ± 1.04**	**39.6 ± 8.7**	**132.02**

^1^ Results reported as the mean value ± standard deviation of the half-maximum concentration in μM; ^2^ Index of selectivity (IS) = LC_50_/EC_50_. Bold data represent compounds with high activity against amastigotes of *L. (V) panamensis*.
